# Flu’s cues: Exploiting host post-translational modifications to direct the influenza virus replication cycle

**DOI:** 10.1371/journal.ppat.1007205

**Published:** 2018-09-20

**Authors:** Anthony R. Dawson, Andrew Mehle

**Affiliations:** 1 Medical Microbiology and Immunology, University of Wisconsin Madison, Madison, Wisconsin, United States of America; 2 Graduate Program in Cellular and Molecular Biology, University of Wisconsin Madison, Madison, Wisconsin, United States of America; Mount Sinai School of Medicine, UNITED STATES

## Introduction

**Fig 1:**
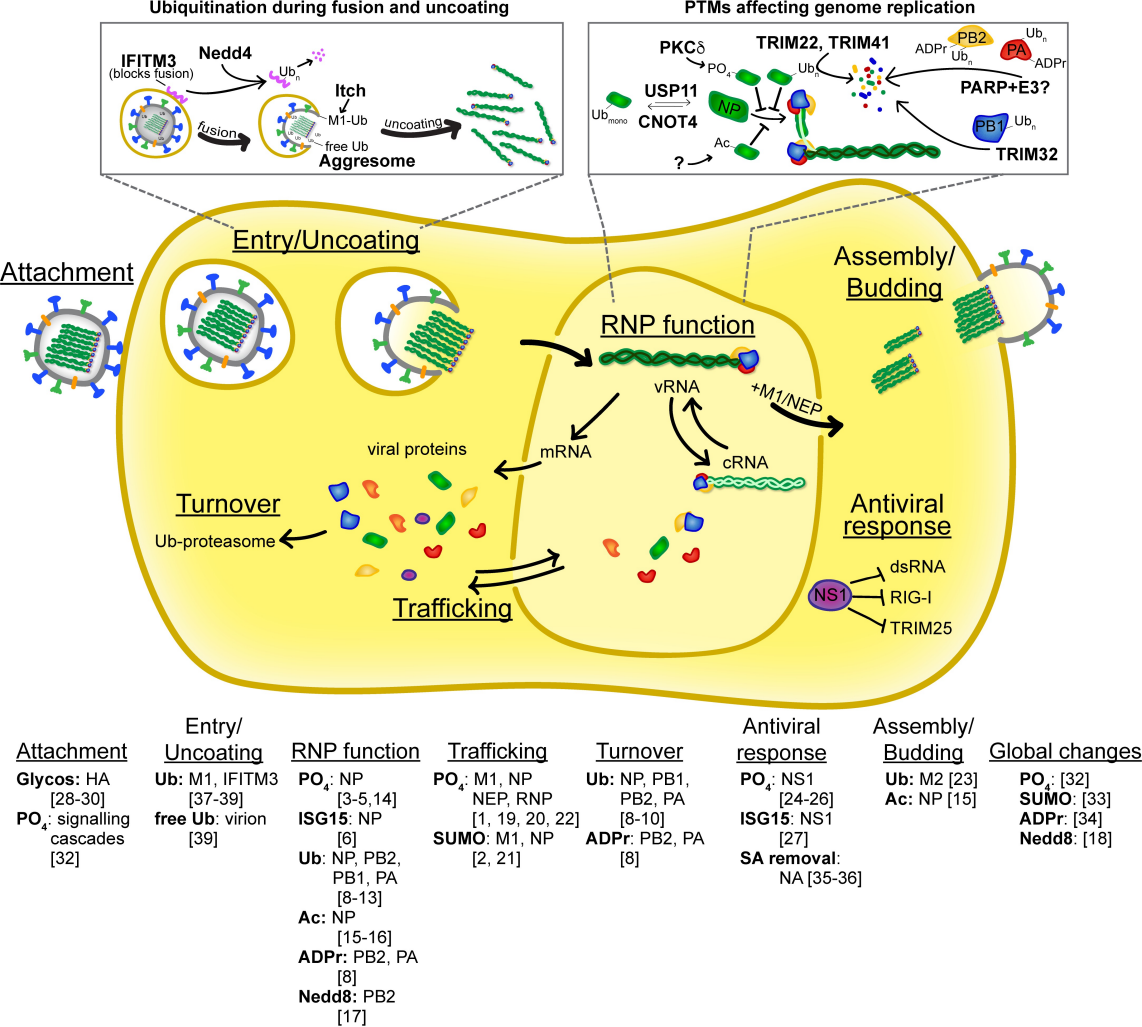
Post-translation control of key steps during the influenza virus replication cycle. Simplified diagram of key steps during the influenza virus life cycle highlighting events that are regulated by PTMs to viral or host proteins. The specific modifications, target proteins, and references are listed for each step. Two processes are highlighted in depth; these examples were chosen because the PTMs and causative host enzymes are known and the modifications have discrete effects on replication. In addition, a large number of PTMs have been identified on viral proteins, but no discrete function has yet been assigned [14]. Ac, acetylation; ADPr, ADP-ribosylation; cRNA, plus-sense genomic RNA; dsRNA, double-stranded RNA; Glycos., N-linked glycosylation; ISG15, ISGylation; Nedd8, neddylation; NP, nucleoprotein; NS1, nonstructural protein 1; PO_4_, phosphorylation; PTM, post-translational modification; RNP, ribonucleoprotein complex; SA, sialic acid; SUMO, SUMOylation; Ub, ubiquitin and ubiquitination; vRNA, minus-sense genomic RNA.

Precise coordination of cellular processes requires prompt specification of protein function in response to various stimuli. This specification includes regulating protein abundance, localization, catalysis, and binding. Post-translational modifications (PTMs) provide cells the plasticity for dynamic and reversible control of protein function. Viral infections provide an exciting lens through which to study PTMs, since PTMs contribute to both cellular responses to infection and viral hijacking of the host. PTMs enhance the already multifunctional nature of viral proteins and offer another level of functional diversity within limited genetic space. Influenza virus protein functions are fine-tuned by diverse types of PTMs, including phosphorylation, ubiquitination, SUMOylation, neddylation, ISGylation, glycosylation, ADP-ribosylation, palmitoylation, and acetylation. All of the major viral proteins are subject to at least one type of PTM. Additionally, as influenza viruses encode no known protein-modifying enzymes, all of these PTMs are mediated by host machinery. Here, we use influenza virus and its proteins as exemplars for how PTMs impact virus replication (Fig 1).

### Do PTMs regulate the function of the influenza ribonucleoprotein complex?

Influenza virus transcribes and replicates its negative single-stranded RNA genome via a virally encoded RNA-dependent RNA polymerase (RdRp). This is performed by the viral ribonucleoprotein complex (RNP), containing genomic RNA encapsidated by the viral nucleoprotein (NP) and bound at both termini by a single heterotrimeric RdRp. While all components of the RNP are post-translationally modified, mechanistic consequences of NP PTMs have been particularly well described. During infection, newly made NP traffics from the cytoplasm into the nucleus and then back to the cytoplasm as either free NP or assembled RNPs. NP trafficking is regulated by both phosphorylation and SUMOylation [1,2]. Phosphorylation of NP at its N-terminal nuclear localization signal inhibits interaction with nuclear import factors, whereas internal NP phosphorylation inhibits interactions with nuclear export factors [1]. SUMOylation appears to be important for nuclear retention, as mutant NPs lacking SUMOylation sites are prematurely exported to the cytoplasm [2]. Viruses encoding NP SUMO-site mutants exhibit profound defects in replication and rapidly revert to wild type.

Nuclear NP oligomerizes along the length of newly synthesized genomic RNA. This process is negatively regulated for both influenza A and B viruses by NP phosphorylation at conserved sites on apposing sides of the homotypic interface [3,4]. Preventing phosphorylation by mutagenesis results in hyperoligomerization of NP, and deleting the responsible host kinase causes severe defects in RNP assembly, function, and viral replication; these data suggest that phosphorylation at these positions is critical for incorporation of NP into nascent RNPs [5]. NP oligomerization during influenza B virus infection is also inhibited by ISGylation, but in this case, the PTM is a possible antiviral response [6].

All RNP components are ubiquitinated, and perturbing global ubiquitination impairs RNP function [7]. In certain contexts, ubiquitination results in protein turnover, especially when polyubiquitin chains are appended [8–11] (Fig 1). However, ubiquitination also plays a regulatory role during infection. NP is dynamically monoubiquitinated and deubiquitinated at lysine 184 (K184) [12,13]. This modification may regulate interaction of NP with genomic RNA to facilitate genome replication. Thus, ubiquitination of RNP proteins plays dual roles, both inhibiting and promoting replication.

Functions of additional RNP PTMs are less well defined. Phosphorylation of the three polymerase subunits—PB1, PB2, and PA—has unknown consequences [14]. PB1, PA, and NP undergo N-terminal acetylation with no specific assigned functions [14]. NP is also acetylated on internal lysine residues [15,16]. Mimicking NP acetylation disrupts the ability of NP to stabilize replication intermediates. Notably, NP is acetylated on K184, the same residue that is monoubiquitinated, suggesting potential cross-talk between different PTMs. The polymerase subunits PB2 and PA are ADP-ribosylated [8]. ADP-ribosylation promotes their ubiquitination and subsequent degradation, providing another example of PTM cross-talk. In an apparent paradox, neddylation of PB2 blocks viral replication [17], yet inhibition of the neddylation pathway also results in poor replication [18]. In sum, RNP PTMs serve both as tunable ways to regulate polymerase function and as antiviral responses that attempt to block replication.

### What do PTMs do for other influenza virus proteins?

In addition to regulating RNPs, PTMs impact genome trafficking and evasion of antiviral responses. RNPs assemble in the nucleus and are exported to the cytoplasm, where they traffic to sites of assembly and budding. Export requires the viral matrix protein (M1) and the nuclear export protein (NEP). Current data support a daisy-chain model in which the RNP interacts with M1, M1 interacts with NEP, and NEP interacts with the cellular export machinery. Phosphorylation of M1 enhances import [19]. M1 phosphoablative mutants remain in the cytoplasm, whereas M1 phosphomimetic mutants or a temperature-sensitive phosphorylation hypermorph are retained in the nucleus [19,20]. All of these mutants exhibit replication defects. Formation of the RNP-M1-NEP daisy chain and its export are affected by M1 SUMOylation and possibly phosphorylation on NEP [14,21]. M1 SUMO-site mutants exhibit decreased interaction with RNPs, resulting in vRNA nuclear export defects and reduced viral titers. Phosphorylation of NEP at several conserved residues adjacent to its nuclear export signal may also control export, although functional analyses indicate that these sites are not essential regulators of NEP function [22]. Whether M1 phosphorylation is reversed concomitant with SUMOylation and nuclear export remains to be determined, but it could represent a system for dynamic control of M1 localization throughout infection. Once exported, RNPs are trafficked to the plasma membrane, where ubiquitination of the viral membrane protein M2 plays a key role in particle assembly and release [23].

PTMs are important for the two main viral proteins that engage the host immune response: the nonstructural protein 1 (NS1) and hemagglutinin (HA). NS1 is the canonical antagonist of innate immune responses. NS1 sequesters dsRNA to avoid detection by host sensors and also antagonizes and directly binds these sensors, including RIG-I and TRIM25. Phosphorylation and ISGylation of NS1 disrupt protein–RNA interactions, while phosphorylation also disrupts protein–protein interactions [24–27]. In this case, the host utilizes PTMs to disarm viral countermeasures. PTMs on the viral glycoprotein HA, however, are exploited by influenza virus to evade immune detection and increase viral spread. The viral glycoprotein HA mediates attachment and entry. HA is the immunodominant viral protein that elicits most of the humoral response from infection. Glycosylation of viral envelope proteins is a well-described mode of immune evasion, and gain or loss of specific N-linked glycosylation sites helps shield influenza HA from antibody recognition and neutralization [28]. HA glycosylation has more recently been shown to increase virulence and fitness after immune escape [29,30]. PTMs of influenza virus proteins vary by host species as well, with potential impacts on replication and pathogenicity. For example, recent data indicate that lower vaccine efficacy results in part from differential HA glycosylation that occurs when viruses are grown in mammalian or avian hosts [31].

### Does influenza virus exploit PTMs to modulate host protein function?

Host cell reprograming during infection often focuses on wholesale changes in gene transcription, such as the induction of interferon-stimulated genes. Remodeling of the host cell might be more broadly considered to also include changes in protein degradation, subcellular localization, and differential activation of cell signaling cascades. Cellular proteins and their PTMs regulate reprogramming events, a feature exploited by influenza virus. Influenza infection results in global changes in PTMs of the host proteome, including triggering kinase cascades [32], reprogramming of cellular SUMOylation [33], stimulation of ADP-ribosylation [34], and activation of the neddylation pathway [18]. PTMs themselves can also be modified. The viral neuraminidase (NA) mediates release of new viral particles by cleaving sialic acid from host glycans. Interestingly, NA removes sialic acid moieties from viral and cellular proteins, including the host cytokine TGF-β, leading to its activation as part of a protective response to infection [35,36].

Influenza viruses indirectly utilize PTMs to co-opt cellular machinery. The ubiquitin machinery plays key roles at multiple steps during entry (Fig 1). Infection-triggered cascades promote ubiquitination of M1 to facilitate release of the incoming virion [37]. Influenza virus entry also relies on the cellular E3 ligase NEDD4 to ubiquitinate and reduce levels of the entry inhibitor IFITM3 [38]. Influenza virions contain nonconjugated ubiquitin chains, which upon entry direct incoming viral cores to the cellular aggresome, where they are efficiently uncoated and associated with the microtubule network for nuclear import of released RNPs [39]. Therefore, the host’s PTM machinery modifies both viral and host proteins, creating a cellular milieu conducive for replication.

### Given what we know about PTMs and influenza virus, what don’t we yet know?

Studies of PTMs have shed light on our understanding of the influenza replication cycle while also raising exciting new questions. Whereas PTMs have been mapped to all viral proteins—with most being modified at multiple sites by diverse PTMs—the host effectors and functional outcomes of most PTMs remain unknown. While some of the PTMs have clearly defined activities (see above), perhaps the largest question is whether all of the modifications discovered so far have functional impacts during infection. Even if all of the PTMs are functionally important, it is possible that some modifications are required only under discrete circumstances but dispensable at other times, making this a more complicated question to address. We also have little appreciation for how these modifications change temporally during infection. It has been proposed that dynamic PTMs could dictate progression of the replication cycle. Indeed, it is the reversible nature of these PTMs that make them attractive mechanisms for dynamic regulation. Yet, how specific PTMs are orchestrated during influenza virus infection and whether this serves to temporally order viral processes has only begun to be explored.

Viral proteins are notoriously multifunctional, and understanding how the varied tasks are separated remains elusive. An exciting possibility is that PTMs parse these different functions by establishing distinct populations of the same viral protein. For example, the separation of viral transcription from genome replication during influenza virus infection is incompletely understood. Viral proteins, small viral RNAs, and various host proteins have all been implicated in biasing polymerase output. The proteins of the viral RNP are modified by multiple PTMs, and PTMs could provide additional mechanisms to establish discrete populations of transcribing versus replicating RNPs.

The roles of PTMs have generally been characterized in isolation, raising the question of how these modifications work in concert. Moreover, since certain amino acid residues can be subject to many different PTMs, a single residue may be competitively or differentially modified over time. Several examples of PTM cross-talk on influenza virus proteins have already been identified [8,12,16], suggesting additional levels of regulatory complexity. Additionally, continuing advances in detection and characterization of PTMs will undoubtedly uncover new facets of influenza virus biology [40]. In summary, influenza viruses utilize PTMs to modulate multiple steps throughout the viral replication cycle. Infection induces global changes in PTMs as well as targeted modifications on specific viral proteins. While many PTMs support viral infection, others are part of antiviral responses. How PTMs change across infection for both host and viral proteins, how PTMs work in concert during replication, and how they impact pathogenicity and host range are exciting and open questions.
